# Establishment and validation of an RNA binding protein-associated prognostic model for ovarian cancer

**DOI:** 10.1186/s13048-021-00777-1

**Published:** 2021-02-07

**Authors:** Chaofan He, Fuxin Huang, Kejia Zhang, Jun Wei, Ke Hu, Meng Liang

**Affiliations:** 1 School of Life Science, Bengbu Medical College, Bengbu, 233030 Anhui People’s Republic of China; 2 Department of Gastroenterology, The First Affiliated Hospital of Bengbu Medical College, Bengbu, 233004 Anhui People’s Republic of China

**Keywords:** RBP, RBP target gene, Ovarian cancer, Risk model, Overall survival

## Abstract

**Background:**

Ovarian cancer (OC) is one of the most common gynecological malignant tumors worldwide, with high mortality and a poor prognosis. As the early symptoms of malignant ovarian tumors are not obvious, the cause of the disease is still unclear, and the patients’ postoperative quality of life of decreases. Therefore, early diagnosis is a problem requiring an urgent solution.

**Methods:**

We obtained the gene expression profiles of ovarian cancer and normal samples from TCGA and GTEx databases for differential expression analysis. From existing literature reports, we acquired the RNA-binding protein (RBP) list for the human species. Utilizing the online tool Starbase, we analyzed the interaction relationship between RBPs and their target genes and selected the modules of RBP target genes through Cytoscape. Finally, univariate and multivariate Cox regression analyses were used to determine the prognostic RBP signature.

**Results:**

We obtained 527 differentially expressed RBPs, which were involved in many important cellular events, such as RNA splicing, the cell cycle, and so on. We predicted several target genes of RBPs, constructed the interaction network of RBPs and their target genes, and obtained many modules from the Cytoscape analysis. Functional enrichment of RBP target genes also includes these important biological processes. Through Cox regression analysis, OC prognostic RBPs were identified and a 10-RBP model constructed. Further analysis showed that the model has high accuracy and sensitivity in predicting the 3/5-year survival rate.

**Conclusions:**

Our study identified differentially expressed RBPs and their target genes in OC, and the results promote our understanding of the molecular mechanism of ovarian cancer. The current study could develop novel biomarkers for the diagnosis, treatment, and prognosis of OC and provide new ideas and prospects for future clinical research.

**Supplementary Information:**

The online version contains supplementary material available at 10.1186/s13048-021-00777-1.

## Introduction

Ovarian cancer is one of the most common gynecological malignancies worldwide [30]. Although OC has a lower incidence than cervical and uterine cancers, it has the highest mortality rate of all gynecological malignancies; ovarian cancer is thus a serious threat to women’s health [35].Unfortunately, as many as 70% of OC patients are not diagnosed with OC until the advanced stage, since early specific signs and symptoms of OC are not obvious and development is rapid; moreover, there is a lack of efficient early diagnostic methods in clinical use [36]. At present, surgery and chemoradiotherapy are the most often used modalities in the treatment of ovarian cancer, but the side effects are severe, and patients’ quality of life of after the operation is seriously decreased [7, 9]. Postoperative cancer recurrence and drug resistance are still difficult problems requiring solutions. Therefore, it is of huge significance to explore specific markers for early diagnosis of ovarian cancer to improve treatment effects and patient prognosis.

Gene expression is regulated at multiple levels in eukaryotes, for example, the epigenetic, transcriptional, and post-transcriptional levels, and so on. Currently, studies on regulation at the transcriptional and translational levels have been relatively thorough and in-depth, but the current understanding of post-transcriptional regulation is still not complete. RNA binding proteins (RBPs) are important components of post-transcriptional modification. They regulate the critical metabolic processes of RNA maturation, transport, stability, and degradation [43]. In all, 1542 genes have been confirmed experimentally to encode RBPs in humans, accounting for about 7.5% of all protein-coding genes. This suggests that RBPs play a significant role in the regulation of gene expression [4, 15]. Because of the omnidirectional and multifunctional regulation of mRNA by RBP, even small changes can cause significant physiological and pathological effects. Previous studies have indicated that cancer, neurodegenerative diseases, and cardiovascular diseases have been proven to be associated with RBP abnormalities [2, 31, 42]. Cell proliferation, differentiation, apoptosis, and other physiological processes are precisely regulated at the post-transcriptional level, which is involved in the development of tumors [24]. Therefore, it is of great significance to understand how RBP regulates gene expression to reveal the mechanism of tumor formation and to search for tumor therapeutic targets.

With the development of science and technology and the in-depth study of RBP by researchers, More and more studies have shown the involvement of RBPs in cancer occurrence. Compared with normal tissue, the expression level of RNA binding protein QKI is significantly lower in gastric cancer tissue. In vitro experiments also confirmed that overexpression of QKI could lead to inhibition of proliferation of gastric cancer cells [5]. Argonaute 2 promotes hepatocellular carcinoma by stabilizing MYC mRNA [45]. In ovarian cancer, SORBS2 inhibits cancer invasion and induces the tumor-inhibiting immune microenvironment by combining WFDC1 with IL-17D 3UTR [46]. LARP1 binding to BCL2 mRNA increases its stability and promotes anti-apoptosis of ovarian cancer cells [21]. Studies have shown that HuR is involved in tumorigenesis by enhancing the stability of target mRNAs such as CCNA1, VEGF, IL8, COX2, etc. [32]. It can be seen that RBP interacts with DNA and proteins to form a complex regulatory network by regulating the life activities of target RNA. Therefore, we acquired ovarian cancer RNA-seq and clinical data from the cancer genome atlas (TCGA) database and downloaded normal tissue RNA-seq data from the Genotype-Tissue Expression (GTEx) database. Through bioinformatics analysis, we obtained differentially expressed RBPs and RBP targets and systematically discussed their potential functions and interactions. Our study identified several OC-related RBPs and RBP target genes to advance our understanding of the molecular mechanisms underlying the development of ovarian cancer. These genes can serve as novel biomarkers for the prevention and diagnosis of ovarian cancer and provide new ideas and perspectives for future clinical research.

## Materials and methods

### Data acquisition

The level 3 gene expression profiles of 379 ovarian cancer patients were downloaded from the TCGA data portal (https://tcga-data.nci.nih.gov/tcga/). All data on 88 normal ovarian tissue samples were obtained from the GTEx data portal (https://gtexportal.org/home/datasets). Perl software was used to process the expression data of the normal group and the tumor group and then merge them. In addition, we obtained the overall survival and phenotype files. The targeting relationship between RNA-binding proteins and their target genes was obtained through the Starbase database (http://starbase.sysu.edu.cn/) [28].

### Identification of differentially expressed genes (DEG)

Differentially expressed RBPs and RBP-Target genes were screened by comparing tumor and normal samples. The DEGs were identified with the R package limma [34], and a heatmap and volcano map were drawn with the R package “pheatmap” and “ggpubr”. A Venn diagram (https://bioinfogp.cnb.csic.es/tools/venny/index.html) was drawn to identify differentially expressed genes. *P* < 0.05 and |Logfold change (FC)| > 1 were considered as the cutoff values of DEGs.

### Functional enrichment analysis

The R package clusterProfiler was used for Kyoto Encyclopedia of Genes and Genomes (KEGG) and Gene Ontology (GO) enrichment analysis [44]. The ontology contains three categories: biological process (BP), molecular function (MF) and cellular component (CC). Enriched GO terms and KEGG pathways results set the threshold *P* < 0.05.

### Construction of an RBP-target gene network

The RBP-Target gene network was retrieved from the Starbase database, and it was reconstructed and analyzed by MCODE and Cytohubba of Cytoscape software. The colors of the nodes in the PPI network reflected the gene expression level |LogFC|; the size of the node indicates the number of proteins that interact with the specified protein.

### Overall survival curve

Survival analysis was performed using survival data from the TCGA database, and a threshold of *P* < 0.05 was set. We verified the overall survival of RBP in the Cox model through different GEO datasets on the Kaplan Meier plotter (https://kmplot.com/analysis/) [17].

### Construction and validation of the cox regression model

We first screened for prognostic RBPs by univariate Cox regression analysis (*P* value < 0.05 was set as the cutoff). Next, prognostic RBP signatures were determined by multivariate Cox regression analysis. In multivariate Cox regression analysis, AIC had a minimum value of 2266.94 as the best cutoff point. The prognosis-related RBP signature is expressed as follows: risk score = (coefficient RBP1 × RBP1 expression) + (coefficient RBP2 × expression of RBP2) + … + (coefficient RBPn × expression RBPn). Finally, the R packages “survival” and “survminer” were used to explore the optimal cutoff for the risk score and draw the survival curve. In particular, the risk curve was performed by grouping the patients into high- and low-risk groups. The R packages “survivalROC” and “timeROC” were used to investigate the time-dependent prognostic value of the gene signature [6, 18].

### Survival analysis

Survival analysis was performed using survival data from the TCGA database and compared by log-rank tests with *P* < 0.05 being statistically significant. Univariate and multivariate cox regression analyses were used to evaluate the survival rates of ovarian patients. The hazard ratio (HR) and 95% confidence interval (CI) were calculated to determine the expression of RBPs associated with overall survival. Results were considered statistically significant at *P* < 0.05.

## Results

### Transcriptomic analysis of differentially expressed genes

**Fig. 1:**
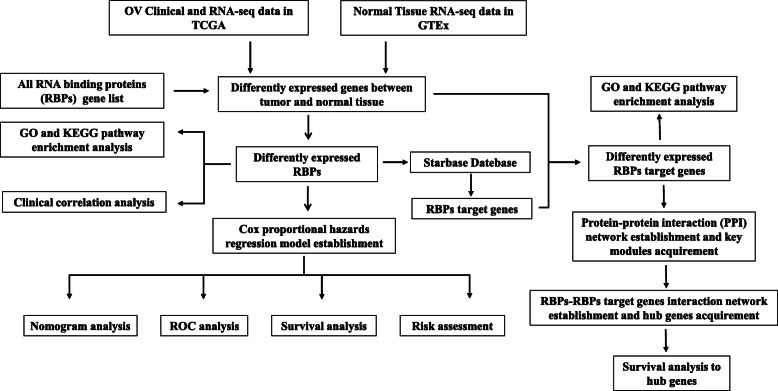
The overall design of the study

**Fig. 2:**
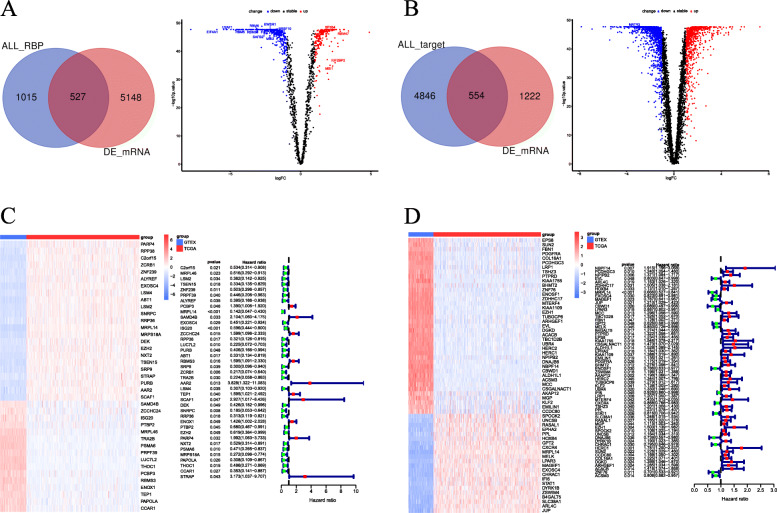
Differential expression analysis and identification of RNA-binding proteins and target genes. **a**, **b** Intersection of abnormally expressed genes and RNA binding proteins and RBP target genes(left), volcano map of abnormally expressed RNA-binding proteins and RBP target genes (right). **c**, **d** Univariate COX regression analysis of heat maps(left) and forest plot(right) of abnormally expressed prognostic RNA-binding proteins and RBP target genes

In this study, we systematically analyzed the critical roles and prognostic value of differentially expressed RBPs and RBP target genes in OC by following the steps described in the Materials and methods section. The workflow of this study is shown in (Fig. 1). We performed a principal component analysis (PCA) with significant extremely differences on the expression profile data of tumor tissues and normal tissues (Figure S1). Based on the thresholds of (*P* < 0.05 and |LogFC| > 1), we identified 527 differentially expressed RBPs (Fig. 2a). At the same time, 554 differentially expressed RBP target genes were obtained, the screening standard of (P < 0.05, |log2FC)| > 2) (Fig. 2b). We used volcanic maps to demonstrate the expression of all RBPs (Fig. 2a), heat maps and forest plot to show statistically significant differences in survival-related RBP expression in tumors and normal tissues (Fig. 2c). Similarly, we show the expression of RBP target genes (Fig. 2b, d).

### Functional enrichment analysis of differentially expressed RBPs and targets

**Fig. 3:**
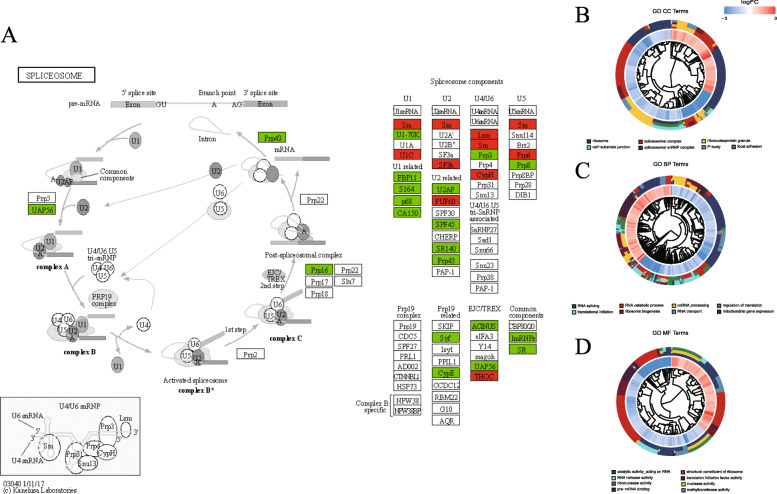
Functional enrichment of abnormally expressed RNA binding proteins. **a** KEGG enrichment of abnormally expressed RNA binding proteins *P* < 0.05. **b**-**d** GO enrichment of abnormally expressed RNA binding proteins

From the 527 differentially expressed RBPs, a total of 8 KEGG pathways were enriched. Among these enriched pathways, all are related to the activity of RNA life, such as the ribosome, the spliceosome, RNA transport, etc. All entries have been listed in (Supplementary Table S1). The visualization result of the spliceosome is shown in (Fig. 3a). Besides, the vital movements of these RBPs were systematically divided into three functional groups: biological process (BP), Molecular Function (MF), and Cellular Component (MF). Analysis results showed that the BP group mainly focused on RNA splicing, RNA catabolic process, ncRNA processing, regulation of translation, translational initiation, ribosome biogenesis, RNA transport, and mitochondrial gene expression (Fig. 3b); the MF group mainly concentrated on structural constituents of the ribosome, ribonuclease activity, catalytic activity acting on RNA, RNA helicase activity, translation initiation factor activity, nuclease activity, methyltransferase activity, and pre-mRNA binding (Fig. 3c); and the CC group mainly involved in the ribosome, the spliceosomal complex, the ribonucleoprotein granule, focal adhesion, the cell-substrate junction, the spliceosomal snRNP complex, and the P-body (Fig. 3d). Similarly, we also carried out enrichment analysis of the differentially expressed 557 target genes. The KEGG enrichment results showed that these genes were closely related to the cell cycle (Supplementary Figure S2). From the RBP target genes, a total of 241 GO terms were enriched. We selected some cancer-related GO terms for visualization, for instance: cell growth, response to oxidative stress, cell-cell junction and protein serine/threonine kinase activity, etc. (Supplementary Table S1).

### RBPs-target interaction network and key module analyses

**Fig. 4:**
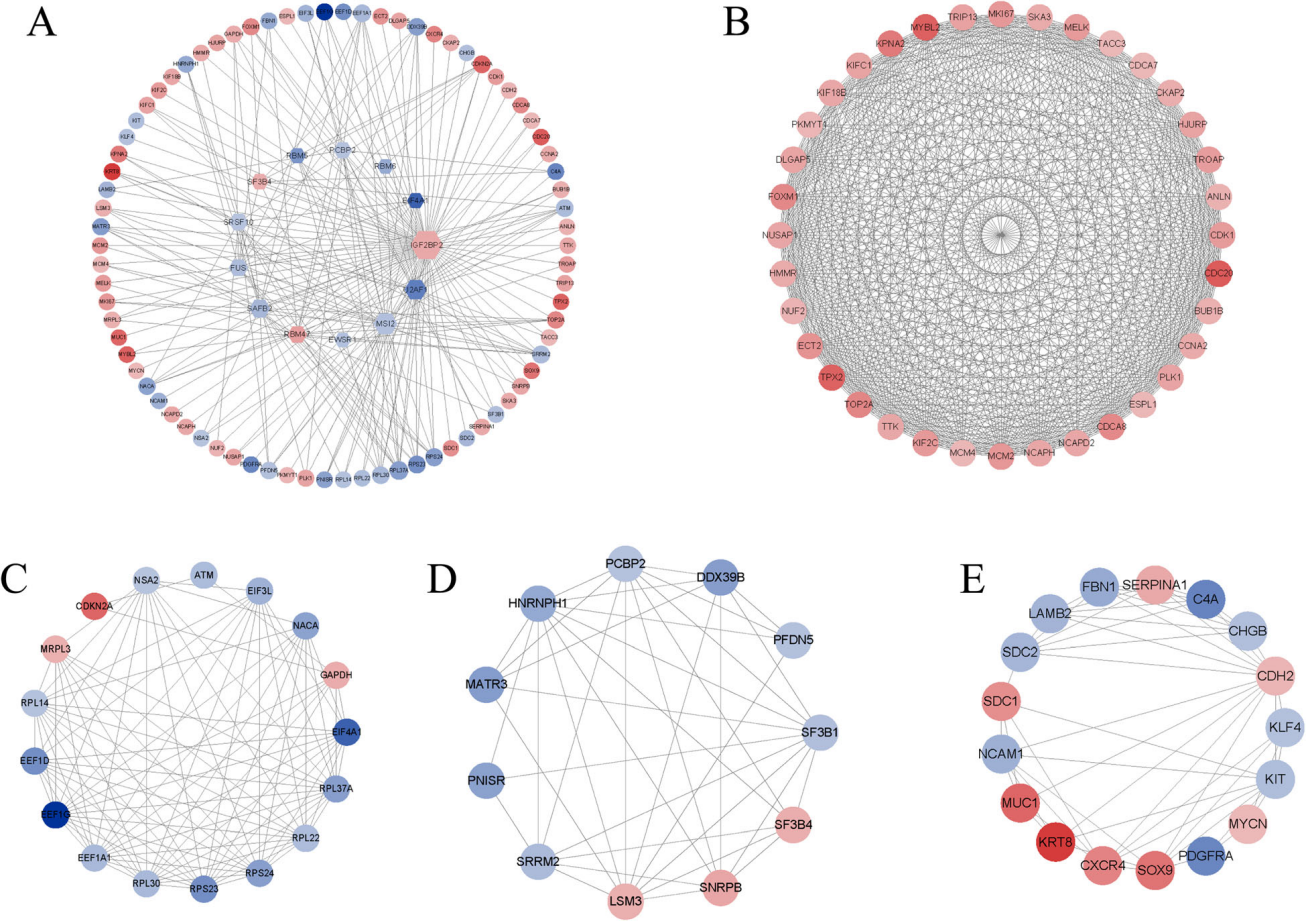
Construction and module analysis of RNA-binding protein and its target gene network **a** Construction of RNA binding protein and its target gene network. **b**-**e** Analysis of RNA binding protein and its target gene module.

Based on the STRING database (https://string-db.org/), we established a PPI network to show the interactions of the 557 target genes. MCODE analysis shows four modules from the core of the network. Module 1 consisted of 36 nodes and 583 edges, module 2 consisted of 17 nodes and 93 edges, module 3 consisted of 11 nodes and 38 edges, and module 4 consisted of 17 nodes and 53 edges. Of the 81 modular genes, 76 target genes were in the top 100 hub genes selected using CytoHubba. It should be noted that the genes in module 1 are extraordinarily closely related to each other. Next, we constructed the relationship network between RBP and RBP target genes in the module genes. We visualized the results (Fig. 4).

### A ten-RBP model and risk score evaluation

**Fig. 5:**
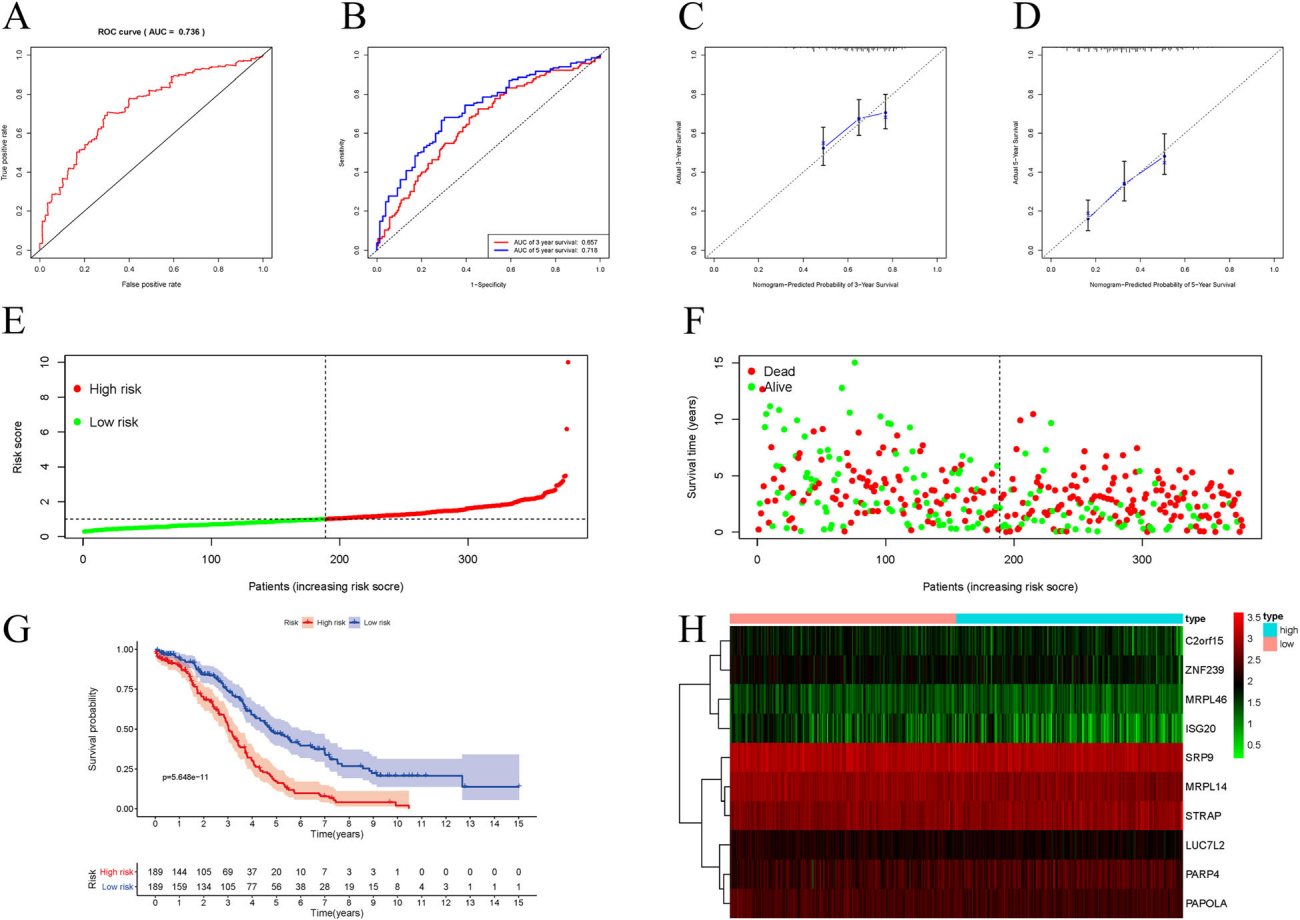
Multivariate Cox regression analysis and model construction. **a** ROC analysis of the ten-RBP model. **b** ROC (3/5-year) analysis of the signature. **c**, **d** Calibration of 3/5-year survival **e**, **f** risk score and survival state analysis for patients in high-risk and low-risk groups by signature. **g** Kaplan-Meier analysis for patients in high-risk and low-risk groups. **h** A heatmap of these 10 RBPs in high-risk and low-risk groups

First, based on the expression of differentially expressed RNA binding protein and survival information acquired from the TCGA database, we obtained a list of RBPs related to prognosis through univariate analysis (Fig. 2c Right). According to our analysis MRPL14, ISG20, etc., showed a significant correlation with prognosis. By applying the COX regression model to single factor prognostic analysis, a stepwise regression approach was used and a ten-prognostic gene model was obtained (*P* < 0.0001). Interestingly, the model was observed that it can be used as an independent predictor of the prognosis of RBPs in OC patients (*P* < 0.05). We generated the formula: risk-score = (− 0.5676) * C2orf15 + (0.6076) * MRPL46 + (− 1.0251) * ZNF239 + (− 1.3075) * MRPL14 + (− 0.7560) * ISG20 + (− 1.2970) * LUC7L2 + (− 1.2124) * SRP9 + (0.5287) * PARP4 + (− 1.9149) * PAPOLA + (1.8874) * STRAP. According to the expression of these RBPs, we calculated the risk value of each case. Based on the median risk score, patients were divided into high- and low-risk groups. We plotted the patient’s survival curve (green for low-risk values, red for high-risk values) and survival state chart (green for survival, red for death) (Fig. 5e, f). Surprisingly, it is evident that the higher the risk, the higher the number of deaths, and the hazard ratio is often associated with poor prognosis.

Further, to more conveniently predict the patient’s 3/5-year survival rate. We used the ten-RBP model to draw a nomogram (Supplementary Figure S3). It is convenient to detect the expression of the ten-RBP and calculate the risk value to predict the corresponding 3/5-year survival rate. These results suggest that the ten-RBP model can be used to assess patients’ prognostic risk. Altogether, we screened multiple-prognosis-related RBPs and constructed a ten-RBP model. This model can achieve the effect of predicting patient risk and the 3/5-year survival rate.

### Validation of the ten-RBP model

Validation was performed, and the ROC curve (AUC = 0.738) analysis of the model showed sensitivity and accuracy (*P* < 0.05). Similarly, for the analysis of the 3/5-year survival rate predicted by the model, we also plotted the R ROC curve and the AUC (> 0.7) analysis showing the sensitivity and accuracy (*P* < 0.05) (Fig. 5a, b). Next, through the calibration curve, we calibrated the nomogram very well and the ten-RBP model performed well in predicting the patients’ 3/5-year survival rate (Fig. 5c, d). Finally, based on the risk groups described above, we found that the high-risk group had a significantly poorer prognosis (*P* < 0.05) (Fig. 5g, h).

### Identification of genes with survival significance

**Fig. 6:**
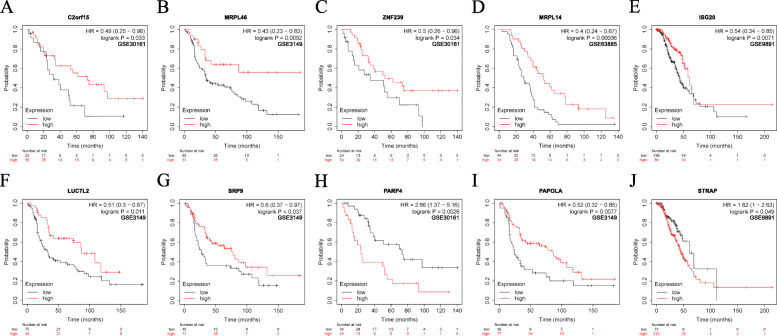
Verification of prognostic genes in GEO datasets. **a** C2orf15, **b** MRPL46, **c** ZNF239, **d** MRPL14, **e** ISG20, **f** LUC7L2, **g** SRP9, **h** PARP4, **i** PAPOLA, and **j** STRAP

**Fig. 7:**
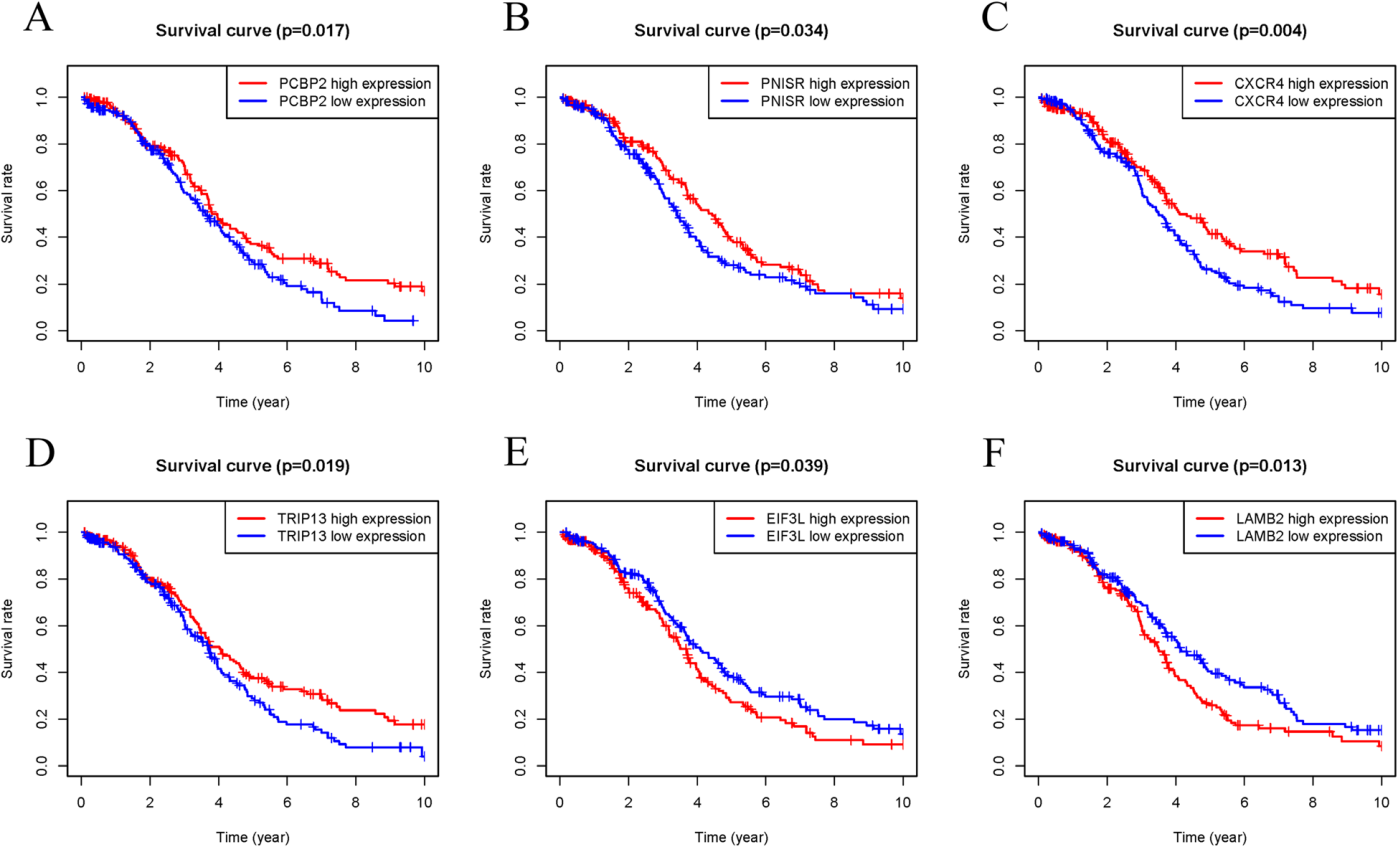
Survival analysis of hub genes of RBP-target. **a** PCBP2, **b** PNISR, **c** CXCR4, **d** TRIP13, **e** EIF3L, and **f** LAMB2

**Fig. 8:**
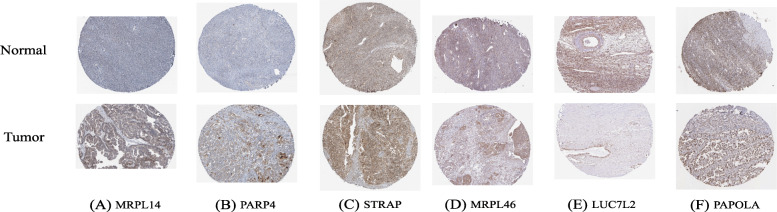
Differential expression of RBPs in the Human Protein Atlas database. **a** MRPL14 (HPA038769), **b** PARP4 (HPA011739), **c** STRAP (HPA027320), **d** MRPL46 (HPA050166), **e** LUC7L2 (HPA051631) and **f** PAPOLA (HPA001788)

We further analyzed the prognostic value of 10 RBPs in the Cox model using the online tool Kaplan-Meier plotter. The results showed that the overall survival of these RBPs in different GEO datasets was basically consistent with the TCGA clinical data analyzed (Fig. 6). In addition, we conducted a batch survival analysis of the different target genes and found that six hub genes (PNISR, CXCR4, TRIP13, EIF3L, LAMB2, and PCBP2) had significant survival significance (Fig. 7). Then we searched for each RBPs in The Human Protein Atlas database (https://www.proteinatlas.org/) and immunohistochemical data showed that the expression levels of these genes (MRPL14, PARP4 and STRAP) were significantly increased, and the antibody staining degree of these genes (MRPL46, LUC7L2 and PAPOLA) was shallow in ovarian cancer tissue (Fig. 8) [39]. This is also consistent with the difference analysis results of RNA-seq data.

In summary, our work builds a ten-RBP model based on the expression of RBPs through univariate and multivariate cox regression analysis. Through this model, the clinical risk and survival prognosis of different patients are well predicted, showing high sensitivity and specificity. The model is expected to be useful in rapid clinical diagnosis of patients and more convenient for providing prognostic information.

## Discussion

Ovarian cancer is a serious threat to women’s health: even with combined treatment, relapse is not uncommon. In the past decades, great breakthroughs have been made in the treatment of ovarian cancer. However, due to the characteristics of drug resistance, easy recurrence and poor prognosis of ovarian cancer, it is not yet possible to completely eradicate the disease [27]. Therefore, it is of great significance to explore the molecular mechanism of ovarian cancer and identify better tumor-specific biomarkers. In this study, we determined 527 differentially expressed RBPs and 554 target genes regulated by partial RBP based on OC data from TCGA and GETx. We systematically analyzed and demonstrated the biological pathways in which these genes are enriched. In addition, a network of interacting relationships between RBPs and target genes was constructed. Finally, Cox regression analysis was performed for differential RBPs to further explore and verify their biological function and clinical significance. We obtained a prognostic risk model for OC based on 10 prognostic related hub RBP genes. Our findings may help to develop new biomarkers for the prevention and diagnosis of ovarian cancer, and provide feasible ideas for clinical research.

The GO functional enrichment analysis showed that RBPs with different expressions were significantly enriched in RNA splicing, RNA catabolic process, ncRNA processing, regulation of translation, structural constituents of the ribosome, translational initiation, ribosome biogenesis, catalytic activity acting on RNA, RNA transport, mitochondrial gene expression, ribonuclease activity, RNA helicase activity, nuclease activity, translation initiation factor activity, methyltransferase activity, pre-mRNA binding, ribosome, spliceosomal complex, ribonucleoprotein granule, focal adhesion, cell-substrate junction, spliceosomal snRNP complex and P-body. Previous studies have confirmed that RNA splicing [13], translation regulation [16], RNA processing [10] and cell adhesion [37] are closely connected with the occurrence and development of malignant tumors. Along with the regulation and metabolism of RNA, RBP plays a major role in post-transcriptional regulation, so we analyzed some target genes of RBP for enrichment analysis. Results of functional pathway enrichment analysis showed that the RBPs targets with different expressions were significantly enriched in cancer-related pathways, for instance: cell growth, response to oxidative stress, cell-cell junction and protein serine/threonine kinase activity, etc. According to our results, several abnormal RBPs are related to spliceosomes. It has been reported that the spliceosome is a new target of carcinogenic stress in MYC-driven cancer, and some components of the spliceosome could provide a novel treatment entry point for MYC-driven cancer [22]. In addition, it has been reported that in cancer, abnormal pre-mRNA splicing plays an important role in the occurrence or development of many human diseases, which are both single-gene and complex [3]. Splicing factors play a critical role in these processes. Various studies have demonstrated that abnormal expression of splicing factors in cancer can cause malignant transformation of cells [38].

Moreover, we created a co-expression network which showed relationships of these abnormally expressed RBPs and their targets and obtained key modules containing 81 key RBP target genes. Many RBPs and target genes have been revealed to play critical roles in the occurrence and progression of tumors. Numerous studies have shown that dysregulation of IGF2BP2 (IGF2 mRNA binding protein 2) can lead to the development of a variety of cancers. The up-regulation of IGF2BP2 activates the PI3K/Akt signaling pathway to promote the growth of pancreatic cancer cells [43]. IGF2BP2 and IGF2BP3 synergistically promote the metastasis of triple-negative breast cancer by promoting the inactivation of progesterone receptors [23]. Knocking down IGF2BP2 significantly reduced the proliferation of ovarian HGSC cell lines [19]. SAFB2 (scaffold attachment factor B2) is a multifunctional protein involved in a variety of cellular processes, known for its role in transcriptional inhibition, and has potential for cancer suppression [20]. Our results showed that SAFB2 was significantly underexpressed in ovarian cancer. Over the past decade, numerous studies have reported that the musashi(MSI) gene, in particular MSI2, is involved in the development of cancer in diverse forms. The specific biological functions of MSI2 and how it is involved in regulating the occurrence of cancer are described in detail in this paper [25].

In addition, based on univariate Cox regression analysis, multivariate Cox regression analysis, and survival analysis, a total of 10 key RBPS related to prognosis were identified, including C2orf15, MRPL46, ZNF239, MRPL14, ISG20, LUC7L2, SRP9, PARP4, PAPOLA, and STRAP. Studies have reported that MRPL46 [1], ISG20 [14], LUC7L2 [40], SRP9 [26], PARP4 [33], and STRAP [29] are involved in the development of tumors. It suggests that these RBP may play a crucial role in ovarian cancer and provide some useful information for the prevention and diagnosis of OC. Next, we established a risk score prognosis model of OC based on TCGA data, and verification results showed that these 10 genes had good diagnostic ability and could select OC patients with poor prognosis. In fact, it is not clear how these 10 RBPs are involved in the molecular mechanism of ovarian cancer formation, and further exploration of their potential mechanisms may provide some new ideas for future research. Subsequently, we established a nomogram to help us more intuitively predict our 3 - and 5-year survival rates. Kaplan Meierplotter was designed to detect the prognostic value of 10 RBPs, and the results were basically consistent with those of the TCGA cohort. In a word, these results suggest that the risk prognosis model based on these 10 RBPs has certain diagnostic and therapeutic value for OC.

Screening methods for ovarian cancer mainly include transvaginal ultrasonography, histological examination and other traditional tumor markers, but these methods have certain clinical application limitations. For example, CA125 (a tumor-associated antigen) assay are less sensitive in the early stages and may be interfered under specific conditions such as menstruation or endometriosi s[11]. It is very important to find new ovarian tumor markers. With the development of science and technology, methods such as gene chip, RNA-seq and proteomics have gradually become the research hotspots in the early diagnosis of ovarian cance r[12]. In addition, biopsies combined with high-throughput techniques can provide more effective information for the diagnosis and treatment of ovarian cancer [8].

## Conclusion

In summary, we analyzed and discussed the biological function and prognostic value of differentially expressed RBPs and target genes in ovarian cancer by bioinformatics methods. These genes may be involved in the development, progression, invasion, metastasis, and progression of ovarian cancer. A prognostic model of 10 RBP-encoding genes and a PPI network based on 13 RBP and their target genes were constructed, which can be used as a reference for the prevention, diagnosis, and treatment of ovarian cancer. To our knowledge, this is the first report to construct an RBP-related prognostic risk model for ovarian cancer. Our findings will help to uncover the pathogenesis of ovarian cancer and develop new OC-specific biomarkers and therapeutic targets. It also provides some novel direction for the diagnosis and treatment of ovarian cancer.

## Supplementary Information

**Additional file 1.**

**Additional file 2.**

**Additional file 3.**

**Additional file 4.**

## Data Availability

The original data supporting the conclusions of this manuscript will be available by the author without undue reservation.
