# Supplementary material for: Establishment and validation of an RNA binding protein-associated prognostic model for ovarian cancer
Source: J Ovarian Res. 2021 Feb 7;14:27. doi: 10.1186/s13048-021-00777-1 (PMC7869493; doi:10.1186/s13048-021-00777-1)
Supplement: Supplementary file 1 — Additional file 1. [file 13048_2021_777_MOESM1_ESM.pdf]

Figure S1

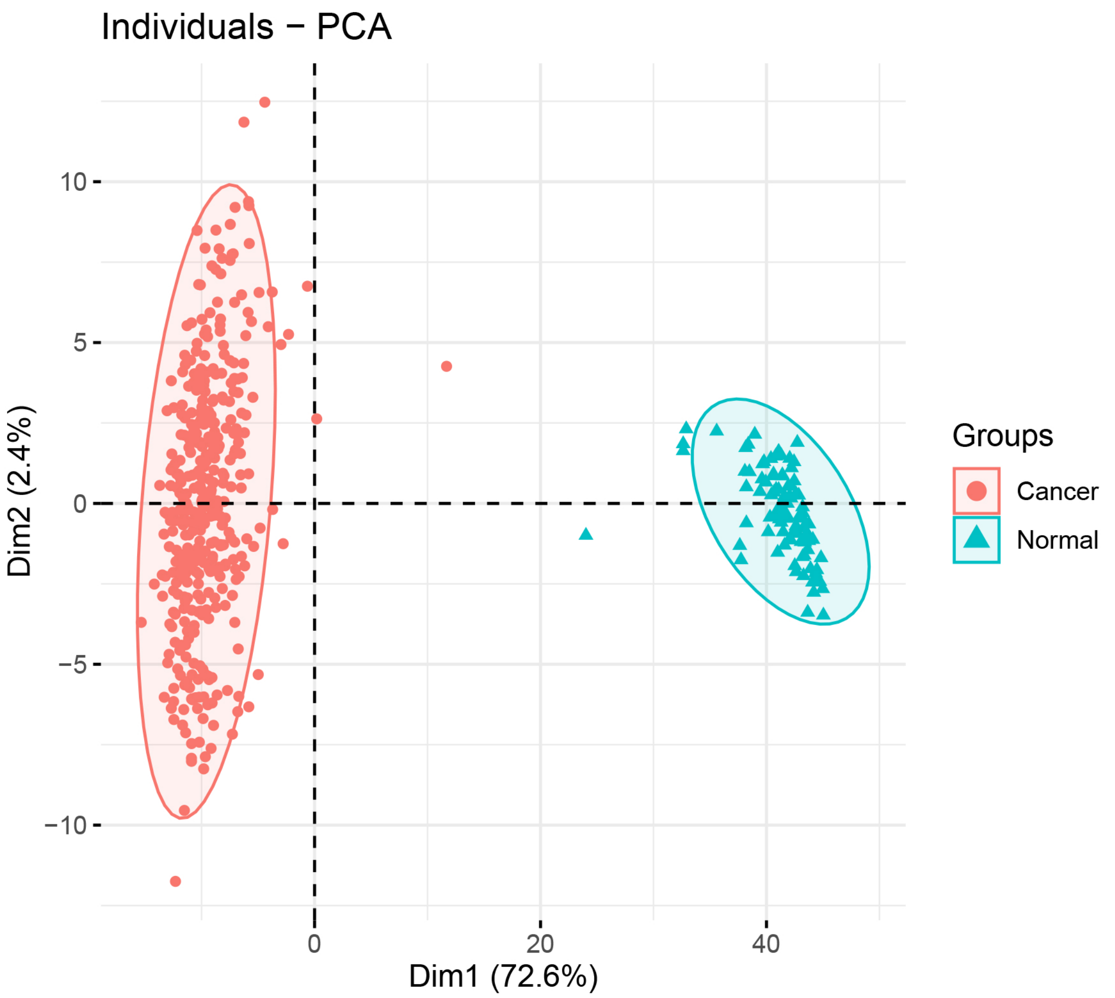

Figure S1: Principal component analysis of ovarian cancer samples in the TCGA-GTEx data set
