# Supplementary material for: Establishment and validation of an RNA binding protein-associated prognostic model for ovarian cancer
Source: J Ovarian Res. 2021 Feb 7;14:27. doi: 10.1186/s13048-021-00777-1 (PMC7869493; doi:10.1186/s13048-021-00777-1)
Supplement: Supplementary file 2 — Additional file 2. [file 13048_2021_777_MOESM2_ESM.pdf]

Figure S2

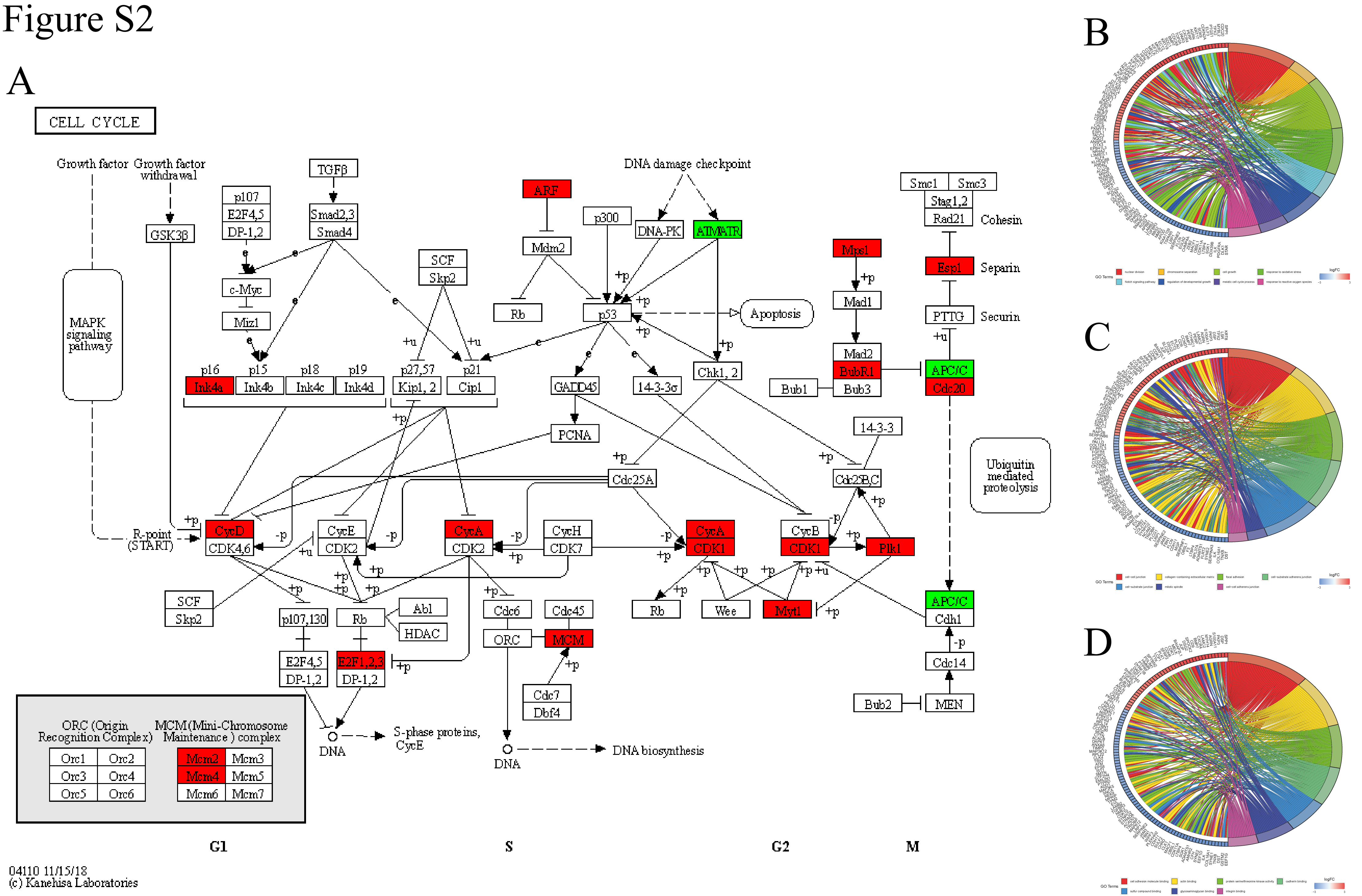

Figure S2: Functional enrichment of differentially expressed RBP-targets. (A) KEGG enrichment of differentially expressed RBP-targets and showed results in cell cycle pathways P<0.05. (B-D) GO enrichment of differentially expressed RBP-targets.
