# Supplementary material for: Establishment and validation of an RNA binding protein-associated prognostic model for ovarian cancer
Source: J Ovarian Res. 2021 Feb 7;14:27. doi: 10.1186/s13048-021-00777-1 (PMC7869493; doi:10.1186/s13048-021-00777-1)
Supplement: Supplementary file 3 — Additional file 3. [file 13048_2021_777_MOESM3_ESM.pdf]

Figure S3

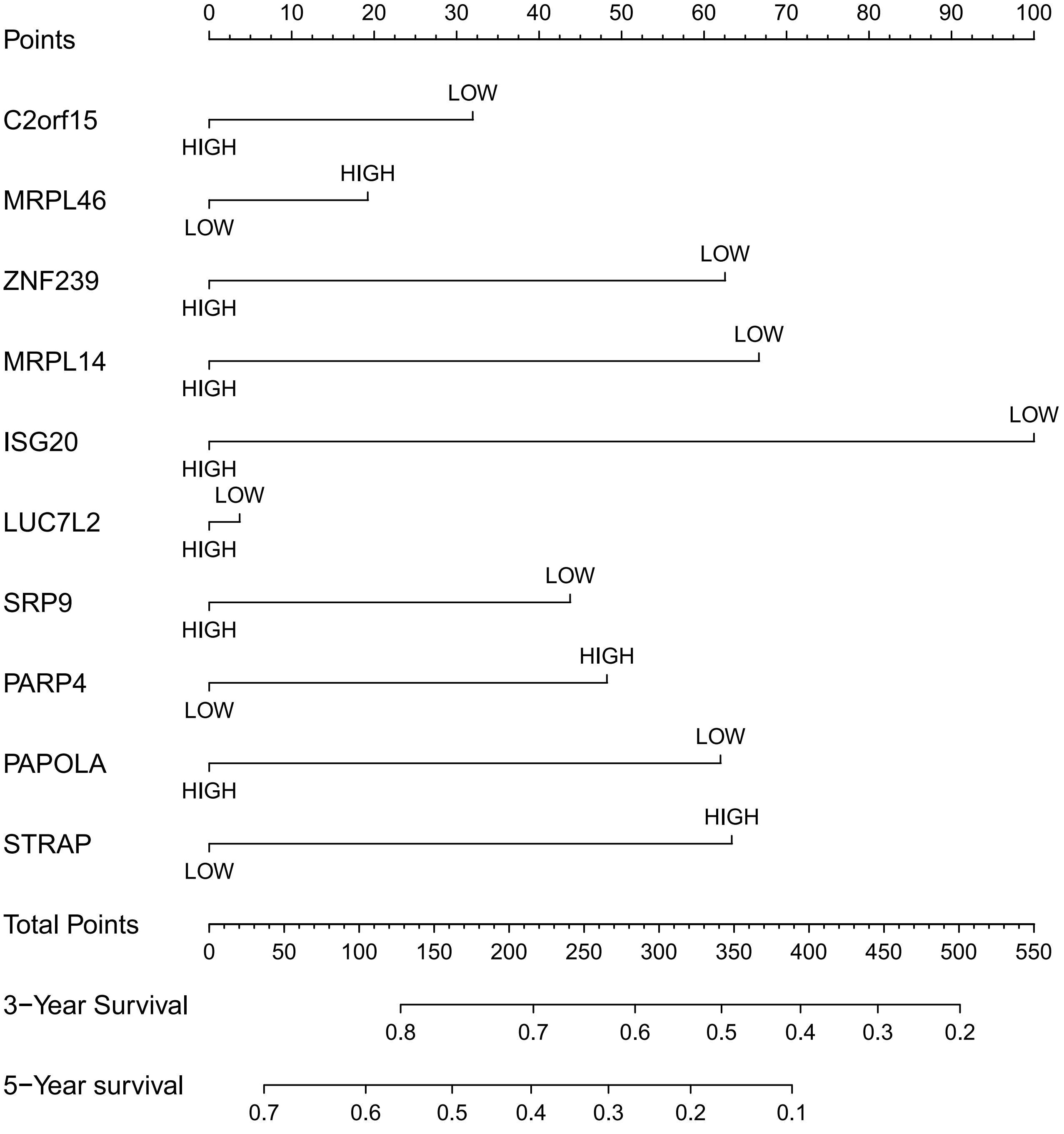

Figure S3: Nomogram for predicting 3-, and 5-year Overall Survival of ovarian cancer patients in the TCGA cohort.
